# Plenary 2 Benefits and challenges of the European Health Data Space

**DOI:** 10.1093/eurpub/ckac128.002

**Published:** 2022-10-25

**Authors:** Organised by: EUPHA, European Commission, Moderators: Iveta Nagyova (EUPHA), Isabel de la Mata (European Commission)

## Abstract

The creation of a European Health Data Space (EHDS) is one of the key components of a strong European Health Union. The objectives of the EHDS are: i) Empower individuals through better digital access to their personal health data; support free movement by ensuring that health data follow people; ii) Unleash the data economy by fostering a genuine single market for digital health services and products; and iii) Set up strict rules for the use of individual’s non-identifiable health data for research, innovation, policy-making and regulatory activities. As such, the EHDS aims to improve and support healthcare delivery within Europe by allowing public health data to be accessible throughout Europe. The EHDS also aims to promote better access and exchange of different types of health data for research and policy purposes. The aim is to have the EHDS up and running in 2025.

The EHDS is expected to bring great benefit, but it also brings challenges related to technology, governance and privacy. The exchange of data at European level means that health data from different sources need to be able to talk to each other. Making the data Findable, Accessible, Interoperable, and Re-usable is key to the success of the EHDS. Moreover, the diversity of Europe’s health information systems need to be taken in account. The EHDS will also have to be transparent to ensure privacy of personal information included in the EHDS.

**Speakers:**

**Fulvia Raffaelli**

European Commission, Brussels, Belgium

**Petronille Bogaert**

Sciensano, Brussels, Belgium

**Irene Schlünder**

TMF, Germany

**Emmanuel Bacry**

Health Data Hub, France

